# The Plant Pathogen *Pseudomonas syringae* pv. *tomato* Is Genetically Monomorphic and under Strong Selection to Evade Tomato Immunity

**DOI:** 10.1371/journal.ppat.1002130

**Published:** 2011-08-25

**Authors:** Rongman Cai, James Lewis, Shuangchun Yan, Haijie Liu, Christopher R. Clarke, Francesco Campanile, Nalvo F. Almeida, David J. Studholme, Magdalen Lindeberg, David Schneider, Massimo Zaccardelli, Joao C. Setubal, Nadia P. Morales-Lizcano, Adriana Bernal, Gitta Coaker, Christy Baker, Carol L. Bender, Scotland Leman, Boris A. Vinatzer

**Affiliations:** 1 Department of Plant Pathology, Physiology, and Weed Science, Virginia Tech, Blacksburg, Virginia, United States of America; 2 CRA-Centro di Ricerca per l′Orticoltura, Sede di Battipaglia, Battipaglia, Salerno, Italy; 3 Virginia Bioinformatics Institute, Virginia Tech, Blacksburg, Virginia, United States of America; 4 Faculty of Computing, Federal University of Mato Grosso do Sul, Campo Grande, Brazil; 5 Biosciences, University of Exeter, Exeter, Devon, United Kingdom; 6 Department of Plant Pathology and Plant – Microbe Biology, Cornell University, Ithaca, New York, United States of America; 7 U. S. Department of Agriculture Agricultural Research Service, Ithaca, New York, United States of America; 8 Department of Computer Science, Virginia Tech, Blacksburg, Virginia, United States of America; 9 Universidad de los Andes, Bogota, Colombia; 10 Department of Plant Pathology, University of California, Davis, California, United States of America; 11 Department of Entomology and Plant Pathology, Oklahoma State University, Stillwater, Oklahoma, United States of America; 12 Department of Statistics, Virginia Tech, Blacksburg, Virginia, United States of America; University of Toronto, Canada

## Abstract

Recently, genome sequencing of many isolates of genetically monomorphic bacterial human pathogens has given new insights into pathogen microevolution and phylogeography. Here, we report a genome-based micro-evolutionary study of a bacterial plant pathogen, *Pseudomonas syringae* pv. *tomato*. Only 267 mutations were identified between five sequenced isolates in 3,543,009 nt of analyzed genome sequence, which suggests a recent evolutionary origin of this pathogen. Further analysis with genome-derived markers of 89 world-wide isolates showed that several genotypes exist in North America and in Europe indicating frequent pathogen movement between these world regions. Genome-derived markers and molecular analyses of key pathogen loci important for virulence and motility both suggest ongoing adaptation to the tomato host. A mutational hotspot was found in the type III-secreted effector gene *hopM1*. These mutations abolish the cell death triggering activity of the full-length protein indicating strong selection for loss of function of this effector, which was previously considered a virulence factor. Two non-synonymous mutations in the flagellin-encoding gene *fliC* allowed identifying a new microbe associated molecular pattern (MAMP) in a region distinct from the known MAMP flg22. Interestingly, the ancestral allele of this MAMP induces a stronger tomato immune response than the derived alleles. The ancestral allele has largely disappeared from today's *Pto* populations suggesting that flagellin-triggered immunity limits pathogen fitness even in highly virulent pathogens. An additional non-synonymous mutation was identified in flg22 in South American isolates. Therefore, MAMPs are more variable than expected differing even between otherwise almost identical isolates of the same pathogen strain.

## Author Summary

Our knowledge of the recent evolution of bacterial human pathogens has increased dramatically over the last five years. By comparison, relatively little is known about recent evolution of bacterial plant pathogens. Here, we analyze a large collection of isolates of the economically important plant pathogen *Pseudomonas syringae* pv. *tomato* with markers derived from the comparison of five genomes of this pathogen. We find that this pathogen likely evolved on a relatively recent time scale and continues to adapt to tomato by minimizing its recognition by the tomato immune system. We find that an allele of the flagellin subunit *fliC* that appeared in the pathogen population for the first time in the 1980s, and which is the most common allele of this gene in North America and Europe today, triggers a weaker tomato immune response than the *fliC* allele found in the 1960s and 1970s. These results not only impact our understanding of pathogen – plant interactions and pathogen evolution but also have important ramifications for disease prevention. Given the speed with which new pathogen strains spread and replace existing strains, limiting the movement of specific strains between geographic regions is critically important, even for pathogens known to have worldwide distribution.

## Introduction

Most taxonomic descriptions of bacterial plant pathogens and studies of their life cycle were performed at a time when it was impossible to classify bacteria precisely. Therefore, it can be difficult to determine whether plant diseases affecting crops in the field today are caused by the same pathogens described in the literature as their causal agents. Moreover, in the absence of precise classification and identification of field isolates, new pathogen variants with increased virulence may spread around the globe unobserved, presenting a potential threat to biosecurity. Furthermore, model plant pathogen strains studied for their molecular interactions with plants in laboratories may not be representative of the pathogens that cause disease in the field and genes required for pathogen success in the field may not even impact bacterial growth or virulence when evaluated under laboratory conditions, which are generally optimized for disease development.

Several human diseases are caused by genetically monomorphic bacterial pathogens that evolved only after the human migration out of Africa. Genome sequencing of multiple strains belonging to each of these pathogens has elucidated their microevolution and their worldwide routes of dispersion. Examples include *Yersinia pestis* [1], *Bacillus anthracis* [2], and *Salmonella* Typhi [3]. Moreover, microevolution of clonal lineages within diverse pathogen species like *Escherichia coli*, *Staphylococcus aureus*, and *Clostridium difficile* have also been unraveled using single nucleotide polymorphisms identified between genomes [4], [5], [6]. Similar studies have yet to be undertaken for plant pathogens. *Pseudomonas syringae* pv. *tomato* (*Pto*) is the causative agent of the bacterial speck disease of tomato (*Solanum lycopersicum*), a disease that occurs worldwide and causes severe reduction in fruit yield and quality, particularly during cold and wet springs, such as occurred in Florida and California in 2010. Three clonal lineages of *Pto* have been previously described based on multilocus sequence typing (MLST): T1, JL1065, and DC3000 [7]. Housekeeping genes of JL1065 and T1 differ in DNA sequence by only 0.4% while DC3000 differs from JL1065 and T1 by 0.9%. JL1065 and T1 were found to be the common pathogenic agents of bacterial speck in the field worldwide. Although DC3000 is a derivative of the pathotype strain of *Pto* and the model pathogen most commonly used to investigate the molecular basis of bacterial speck disease [8], this lineage is only rarely found on tomato [7]. Comparing genomes of multiple isolates of the *P. syringae* pv. *tomato* (*Pto*) T1 lineage and performing a Single Nucleotide Polymorphism (SNP) analysis of a large collection of T1-like strains, we attempt here for the first time to unravel the microevolution and global spread of a bacterial plant pathogen.

## Results/Discussion

### T1-like strains are the most common Pto strains today

**Figure 1:**
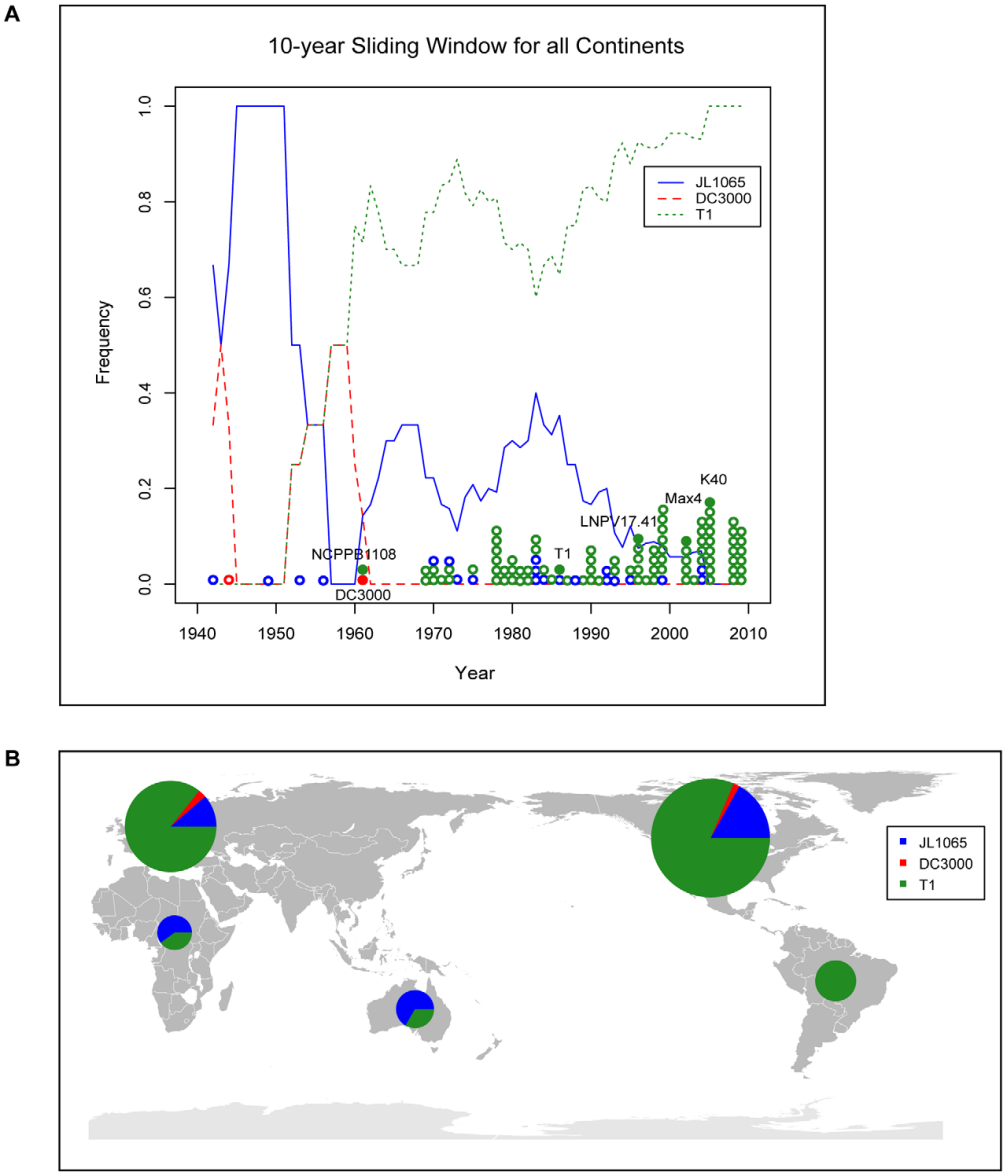
Strains of the T1-lineage have been the most common *Pto* strains since the 1960s and are present in all continents from which *Pto* strains were isolated. (A) The lines indicate the frequency of T1-, JL1065-, and DC3000-like strains over time using a 10-year sliding window with a one-year step. Circles represent individual isolates and are placed in the graph in correspondence to the exact year at which isolates were collected. Full circles indicate isolates of which the genomes have been sequenced. (B) World map with pie charts showing ratio of T1-, JL1065-, and DC3000-like strains for the continents from which *Pto* strains have been analyzed. Pie size is proportional to the total number of strains considered per continent.

Extending our previous MLST analysis [7] to 112 *Pto* isolates collected worldwide between 1942 and 2009 (Table 1) we confirmed that T1 is the most common *Pto* lineage, followed by JL1065 and DC3000. In fact, among all analyzed isolates only two DC3000-like strains and twenty-one JL1065-like strains were found while 89 isolates belonged to the T1 lineage. When plotting strain frequency over time (Figure 1A) and considering geographic origin of strains (Table 1 and Figure 1B), we observed an intriguing trend: DC3000-like and JL1065-like strains were the only *Pto* strains isolated until 1961 when the first T1-like strain was collected in the UK. T1-strains then quickly increased in frequency becoming the most common *Pto* lineage. Some JL-1065 strains were still isolated in the 1980s and 1990s but all strains in our collection isolated in Europe and North America after 1999 belong to the T1 lineage.

**Table 1:** *Pto* isolates used in this study sorted first by MLST genotype (GT) and then by year of isolation.

name	Country	Year	MLST GT	SNP GT^1^	HopM1 allele	obtained from	reference
ICMP 4325	Canada	1944	DC3000	-	DC3000	C. Bender, Oklahoma State U., USA	[49]
DC3000	UK	1961	DC3000	-	DC3000	J. Greenberg, U. of Chicago, USA	[8]
NCPPB 1008	USA	1942	JL1065	-	JL1065	C. Bender, Oklahoma State U., USA	[50]
CFBP 1696	Denmark	1949	JL1065	-	JL1065	CFBP, France	this paper
NCPPB 880	Yugoslavia	1953	JL1065	-	JL1065	C. Bender, Oklahoma State U., USA	[51]
ICMP 2846	USA	1956	JL1065	-	JL1065	C. Bender, Oklahoma State U., USA	[49]
CFBP 1319	Switzerland	1970	JL1065	-	JL1065	CFBP, France	this paper
CFBP 1785	Australia	1972	JL1065	-	JL1065	CFBP, France	this paper
ICMP 3647	Australia	1973	JL1065	-	JL1065	C. Bender, Oklahoma State U., USA	[52]
ICMP 4355	Australia	1975	JL1065	-	JL1065	C. Bender, Oklahoma State U., USA	[53]
JL1065	USA	1983	JL1065	-	JL1065	R. Jackson, U. Reading, UK	[52]
BS118	USA	1983	JL1065	-	JL1065	C. Bull, USDA ARS, Salinas, USA	this paper
BS120	USA	1983	JL1065	-	JL1065	C. Bull, USDA ARS, Salinas, USA	this paper
DC84-1	Canada	1984	JL1065	-	JL1065	D. Cuppels, Agrifood Canada	[54]
PST26L	S. Africa	1986	JL1065	-	JL1065	D. Cuppels, Agrifood Canada	[54]
CFBP 3728	Yemen	1988	JL1065	-	JL1065	CFBP, France	this paper
PT 28	Mexico	1992	JL1065	-	JL1065	J. Jones, U. of Florida, USA	this paper
PT 29	Mexico	1992	JL1065	-	JL1065	J. Jones, U. of Florida, USA	this paper
CPST 147	Czek Rep.	1993	JL1065	-	JL1065	C. Bender, Oklahoma State U., USA	[55]
56	USA	1995	JL1065	-	JL1065	G. Coaker, UC Davis, USA	this paper
Pst field 8	USA	1999	JL1065	-	JL1065	A. Bernal, U. de los Andes, Colombia	this paper
KS 112 lr	Tanzania	2004	JL1065	-	JL1065	M. Zaccardelli, CRA ORT, Italy	[56]
KS 097 lr	Tanzania	2004	JL1065	-	JL1065	M. Zaccardelli, CRA ORT, Italy	[56]
NCPPB 1108	UK	1961	T1	NCPPB1108	1108	D. Cuppels, Agrifood Canada	[54]
CNBP 1318	Switzerland	1969	T1	CFBP1318	T1	D. Cuppels, Agrifood Canada	[54]
NCPPB 2424	Switzerland	1969	T1	CFBP1318	T1	C. Bender, Oklahoma State U., USA	[51]
CFBP 1321	Switzerland	1970	T1	CFBP1318	T1	CFBP, France	this paper
CFBP 1322	Switzerland	1970	T1	CFBP1318	T1	CFBP, France	this paper
CFBP 1323	France	1971	T1	NCPPB1108	PT21	CFBP, France	[51]
CFBP 1426	France	1972	T1	CFBP1318	T1	CFBP, France	this paper
CFBP 1427	France	1972	T1	CFBP1318	T1	CFBP, France	this paper
DAR 31861	Australia	1975	T1	NCPPB1108	PT21	C. Bender, Oklahoma State U., USA	[51]
PT 14	USA	1978	T1	PT14	T1	J. Jones, U. of Florida, USA	this paper
SM78-1	USA	1978	T1	T1	T1	D. Cuppels, Agrifood Canada	[54]
DAR 30555	Australia	1978	T1	PT14	T1	C. Bender, Oklahoma State U., USA	[51]
CFBP 1916	Canada	1978	T1	PT14	T1	CFBP, France	this paper
CFBP 1918	Canada	1978	T1	PT14	T1	CFBP, France	this paper
CFBP 2545	France	1978	T1	CFBP2545	T1	CFBP, France	this paper
487	Greece	1979	T1	CFBP1318	T1	D. Cuppels, Agrifood Canada	[54]
CFBP 6876	France	1979	T1	CFBP2545	T1	CFBP, France	this paper
PST 6	Canada	1980	T1	PT14	T1	T. Denny U. of Georgia, USA	this paper
PT 18	USA	1980	T1	PT14	T1	T. Denny U. of Georgia, USA	this paper
AV80	USA	1980	T1	T1	T1	D. Cuppels, Agrifood Canada	[54]
B181	USA	1981	T1	PT14	T1	T. Denny U. of Georgia, USA	[51]
DCT6D1	Canada	1981	T1	PT14	T1	D. Cuppels, Agrifood Canada	[54]
188B	Canada	1982	T1	T1	T1	D. Cuppels, Agrifood Canada	[54]
BS117	USA	1982	T1	PT14	T1	C. Bull, USDA ARS, Salinas, USA	this paper
PT 17	USA	1983	T1	T1	T1	T. Denny U. of Georgia, USA	this paper
PT 2	USA	1983	T1	PT14	T1	T. Denny U. of Georgia, USA	this paper
CFBP 4408	France	1984	T1	CFBP1318	T1	CFBP, France	this paper
RG4	Venezuela	1985	T1	CFBP1318	T1	C. Bender, Oklahoma State U., USA	[51]
T1	Canada	1986	T1	T1	T1	T. Denny U. of Georgia, USA	[10]
CFBP 4409	France	1987	T1	CFBP1318	T1	CFBP, France	this paper
DC89-4H	Canada	1989	T1	PT14	T1	D. Cuppels, Agrifood Canada	[54]
PT 21	USA	1990	T1	NCPPB1108	PT21	J. Jones, U. of Florida, USA	this paper
PT 23	USA	1990	T1	LNPV17.41	T1	J. Jones, U. of Florida, USA	[57]
PT 25	USA	1990	T1	LNPV17.41	T1	J. Jones, U. of Florida, USA	this paper
PT 26	USA	1990	T1	NCPPB1108	PT21	J. Jones, U. of Florida, USA	this paper
OMP-BO 407/91	Italy	1991	T1	LNPV17.41	T1	M. Zaccardelli, CRA ORT, Italy	[58]
PT 32	USA	1993	T1	LNPV17.41	T1	J. Jones, U. of Florida, USA	this paper
CPST 236	Slovakia	1993	T1	PT14	T1	C. Bender, Oklahoma State U., USA	[55]
IPV-CT 28.31	Italy	1995	T1	IPV-CT28.31	T1	M. Zaccardelli, CRA ORT, Italy	this paper
IPV-BO 2973	Italy	1996	T1	PT14	T1	M. Zaccardelli, CRA ORT, Italy	[58]
LNPV 17.41	France	1996	T1	LNPV17.41	T1	M. Zaccardelli, CRA ORT, Italy	this paper
OMP-BO 443.1/96	Italy	1996	T1	PT14	T1	M. Zaccardelli, CRA ORT, Italy	[58]
A9	USA	1996	T1	LNPV17.41	T1	M. Davis, UC Davis, USA	[59]
CFBP 5420	Macedonia	1996	T1	LNPV17.41	T1	CFBP, France	this paper
407	USA	1997	T1	LNPV17.41	T1	M. Davis, UC Davis, USA	[59]
LNPV 18.76	France	1998	T1	LNPV17.41	T1	M. Zaccardelli, CRA ORT, Italy	this paper
838-1	USA	1998	T1	LNPV17.41	T1	M. Davis, UC Davis, USA	this paper
315	USA	1998	T1	CA315	PT21	G. Coaker, UC Davis, USA	[59]
316	USA	1998	T1	LNPV17.41		G. Coaker, UC Davis, USA	[59]
Pst field 1	USA	1999	T1	LNPV17.41	T1	A. Bernal, U. de los Andes, Colombia	this paper
Pst field 2	USA	1999	T1	LNPV17.41	T1	A. Bernal, U. de los Andes, Colombia	this paper
Pst field 3	USA	1999	T1	LNPV17.41	T1	A. Bernal, U. de los Andes, Colombia	this paper
Pst field 4	USA	1999	T1	LNPV17.41	T1	A. Bernal, U. de los Andes, Colombia	this paper
Pst field 5	USA	1999	T1	LNPV17.41	T1	A. Bernal, U. de los Andes, Colombia	this paper
Pst field 6	USA	1999	T1	PT14	T1	A. Bernal, U. de los Andes, Colombia	this paper
B98 or 57	USA	1999	T1	LNPV17.41		G. Coaker, UC Davis, USA	this paper
Max 1	Italy	2002	T1	LNPV17.41	T1	M. Zaccardelli, CRA ORT, Italy	[7]
Max 4	Italy	2002	T1	LNPV17.41	T1	M. Zaccardelli, CRA ORT, Italy	this paper
Max 5	Italy	2002	T1	LNPV17.41	T1	M. Zaccardelli, CRA ORT, Italy	this paper
Max 6	Italy	2002	T1	LNPV17.41	T1	M. Zaccardelli, CRA ORT, Italy	this paper
ISCI 181	Italy	2002	T1	IPV-CT28.31	T1	M. Zaccardelli, CRA ORT, Italy	this paper
ISCI 78	Italy	2003	T1	LNPV17.41	T1	M. Zaccardelli, CRA ORT, Italy	this paper
KS P 53	Tanzania	2004	T1	KSP53	T1	M. Zaccardelli, CRA ORT, Italy	[56]
KS 127 M	Tanzania	2004	T1	KSP53	T1	M. Zaccardelli, CRA ORT, Italy	[56]
ISCI 284	Italy	2004	T1	IPV-CT28.31	T1	M. Zaccardelli, CRA ORT, Italy	this paper
ISCI 286	Italy	2004	T1	IPV-CT28.31	T1	M. Zaccardelli, CRA ORT, Italy	this paper
ISCI 269	Italy	2004	T1	IPV-CT28.31	T1	M. Zaccardelli, CRA ORT, Italy	this paper
K40	USA	2005	T1	LNPV17.41	T1	C. Waldenmeier, VT, USA	this paper
K41	USA	2005	T1	LNPV17.41	T1	C. Waldenmeier, VT, USA	this paper
K100	USA	2005	T1	LNPV17.41	T1	C. Waldenmeier, VT, USA	this paper
838-4	USA	2005	T1	LNPV17.41	T1	G. Coaker, UC Davis, USA	[59]
838-16	USA	2005	T1	LNPV17.41	T1	G. Coaker, UC Davis, USA	[59]
836-2	USA	2005	T1	LNPV17.41	T1	G. Coaker, UC Davis, USA	[59]
838-8	USA	2005	T1	LNPV17.41	T1	G. Coaker, UC Davis, USA	[59]
838-9	USA	2005	T1	LNPV17.41	T1	G. Coaker, UC Davis, USA	[59]
838-6	USA	2005	T1	LNPV17.41	T1	G. Coaker, UC Davis, USA	[59]
1020	USA	2008	T1	LNPV17.41	T1	E. Bush, VT, USA	this paper
1021	USA	2008	T1	LNPV17.41	T1	E. Bush, VT, USA	this paper
410	USA	2008	T1	LNPV17.41	T1	G. Coaker, UC Davis, USA	[59]
16	USA	2008	T1	LNPV17.41	T1	G. Coaker, UC Davis, USA	[59]
20	USA	2008	T1	LNPV17.41	T1	G. Coaker, UC Davis, USA	[59]
21	USA	2008	T1	LNPV17.41	T1	G. Coaker, UC Davis, USA	[59]
22	USA	2008	T1	LNPV17.41	T1	G. Coaker, UC Davis, USA	[59]
338	Colombia	2009	T1	Colombia338	T1	A. Bernal, U. de los Andes, Colombia	this paper
196	Colombia	2009	T1	Colombia338	T1	A. Bernal, U. de los Andes, Colombia	this paper
198	Colombia	2009	T1	Colombia198	T1	A. Bernal, U. de los Andes, Colombia	this paper
199	Colombia	2009	T1	Colombia338	T1	A. Bernal, U. de los Andes, Colombia	this paper
201	Colombia	2008	T1	Colombia198	T1	A. Bernal, U. de los Andes, Colombia	this paper
204	Colombia	2009	T1	Colombia198	T1	A. Bernal, U. de los Andes, Colombia	this paper

1 SNP genotype sequences are listed in Table S4. SNP genotypes are only listed for T1-like strains (i.e., strains with MLST genotype T1).

### Genomes of five T1-like strains are extremely similar to each other

To investigate the recent evolution and virulence mechanisms of the T1 lineage, we obtained draft genome sequences using Illumina technology [9] of four T1-like strains in addition to the already sequenced genome of strain T1 [10], which was collected in Canada in 1986. These four newly sequenced strains are: NCPPB1108 collected in the UK in 1961, LNPV17.41 collected in France in 1996, Max4 collected in Italy in 2002, and K40 isolated in the USA in 2005. These strains were chosen because they represent the diversity of our strain collection in regard to time of isolation and geographic location. The genomes of NCPPB1108, LNPV17.41, and K40 were assembled and submitted to the NCBI genome database (NZ_ADGA00000000, ADFZ00000000, NZ_ADFY00000000), annotated, and predicted protein repertoires were compared with other *P. syringae* genomes. The genome of Max4 was neither submitted to NCBI nor annotated owing to significantly higher fragmentation relative to the other three genomes. A summary description of genomes can be found in Table 2 and predicted protein repertoires can be compared with additional *P. syringae* genomes online at genome.ppws.vt.edu .

**Table 2:** Summary of *Pto* draft genome sequences.

Strain	Number of Contigs	N50	Largest Contig Size (bp)	Total Length (bp)	Illumina (X)^1^
NCPPB1108	304	46775	153603	6182607	42.6
K40	582	25354	104626	6254280	32.4
LNPV17.41	350	62385	239369	6157021	74.7
Max4	1176	12264	53242	6209056	27.5^2^

1 Coverage was calculated based on total length of all reads used in each assembly.

2 Assembled with a combination of both 454 and Illumina sequences (indicated coverage is based on Illumina reads only).

Sequencing reads were aligned against the DC3000 genome and 11,145 high confidence single nucleotide polymorphisms (SNPs) were identified between DC3000 and the five T1-like genomes using the program MAQ [11]. However, only a total of 157 SNPs were identified between any of the five T1-like strains, underscoring the close relationship among these strains (Table S1). To validate the identified SNPs we also used a second approach. This time we called SNPs between the five T1-like genomes using the T1 genome as reference for alignment, used less stringent criteria, but limited SNP identification to *P. syringae* core genome genes (see details in regard to the differences between Maq settings used in the two approaches in the Materials and Methods section). Limiting SNP identification to the core genome allowed reliable SNP calls applying less stringent settings since genes in the core genome are present only in single copy, thus avoiding misalignment of reads typical with multigene families. 265 SNPs (listed in Table S2) were identified in this way. Twenty-three of these SNPs were re-sequenced from PCR products using Sanger sequencing and all were confirmed (data not shown) giving us confidence in the reliability of this second approach. Since the total length of the core genome used for SNP identification in the second approach was 3,543,009 nt and the identified number of SNPs distinguishing pairs of genomes was found to be between 53 and 183 (based on the SNPs listed in Table S2), the five T1-like core genomes were determined to have pair-wise genetic distances between 0.000017 and 0.000098. This clearly shows that *Pto* is a genetically monomorphic pathogen similar to, for example, *Yersinia pestis* or *Salmonella* Typhi, both of which evolved only subsequent to human migration out of Africa [12]. However, it is challenging to even estimate an approximate divergence time for the five sequenced T1-like strains since a yearly mutation rate has not yet been determined for any plant associated bacterium and data from the five genomes sequenced here are not sufficient to reliably infer a mutation rate based on the sequenced strains themselves and their time of isolation. Nonetheless, we attempted to get a rough estimate of divergence time assuming a minimum mutation rate of 3.4×10^−9^ per base pair per year as estimated for bacteria based on the *E. coli* and *Salmonella enterica* split [13] and a maximum mutation rate of 5×10^−6^ per bp per year, which is similar to the maximum clock rates recently inferred for a clonal methicillin resistant *S. aureus* (MRSA) lineage [14] and for *Helicobacter pylori* [15] and similar to a maximum clock rate assumed previously for the plant pathogen *Clavibacter michiganensis* subsp. *sepedonicus* [16]. We then used the programs IMa2 [17], [18] and BEAST [19] to calculate divergence times for each pair of strains. The obtained results suggest divergence times of around thousand years or less using the maximum mutation rate (Table S3) or around one million years using the minimum mutation rate. However, [13]considering that some of the T1-like genomes have a genetic distance from each other similar to that of the MRSA isolates analyzed by Nübel and colleagues [14] for which a divergence time of only 20 years was inferred, we believe that T1-like strains have likely evolved from their most recent common ancestor after the domestication of tomato, which must have occurred sometime before the 15^th^ century when tomatoes were first brought from Mexico to Europe [20]. To obtain a more reliable estimate of divergence times the yearly mutation rate for plant pathogens will need to be inferred in the future based on the genomes of many more strains isolated in different years from a geographic area, where the approximate year of a single recent introduction is known, as is the case for example for *P. syringae* pv *aesculi* recently introduced into the United Kingdom [21].

**Figure 2:**
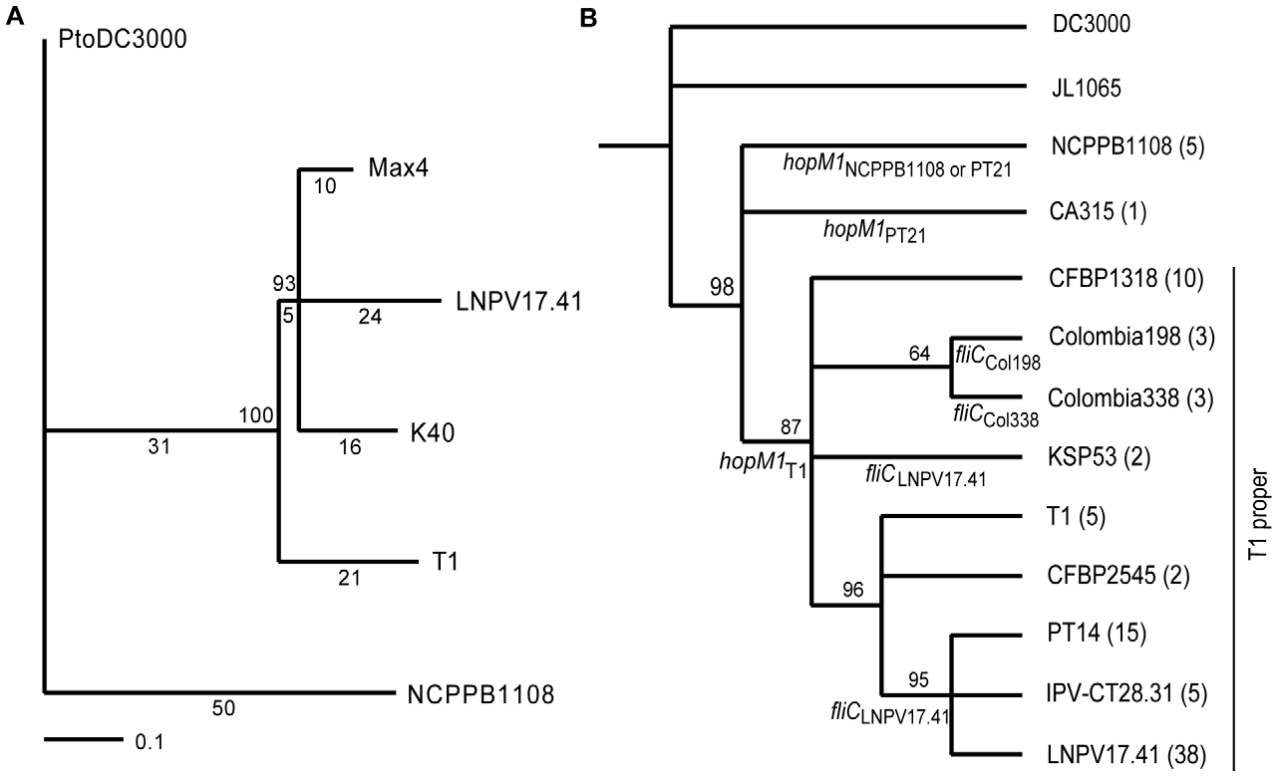
Phylogenetic trees based on SNPs reveal the evolutionary relationship between T1-like *Pto* strains. (A) Maximum likelihood tree based on 157 high quality SNPs identified between five genomes of T1-like strains by aligning Illumina sequencing reads against the DC3000 genome (which was used as an outgroup). The number of SNPs/branch are indicated underneath each branch and bootstrap values are indicated above each branch. A neighbor-joining tree and maximum parsimony tree were also constructed and had identical topology. (B) Maximum likelihood tree based on twenty-four SNPs identified between DC3000-like, JL1065, and T1-like strains in the housekeeping genes *rpoD*, *pgi*, and *gapA* and based on 16 SNPs identified between T1-like strains in 11 fragments of *P. syringae* core genome genes (highlighted in Table S2). Bootstrap values are indicated above each branch and number of strains that belong to each genotype are indicated in parenthesis. Clade-specific *fliC* and *hopM1* alleles are indicated below branches. The clade corresponding to strains called “T1-proper” in the main text is labeled as such. A maximum parsimony tree was also constructed and had identical topology. Since branch lengths of the tree are influenced by our selection of SNP loci, branch lengths are not scaled to evolutionary changes. Table 1 lists strains belonging to each genotype and Table S4 lists DNA sequences of each genotype.

A phylogenetic tree was then constructed based on the SNPs indentified by aligning sequencing reads of the five T1-like strains against the DC3000 genome (Figure 2A). DC3000 was used as outgroup but only SNPs that distinguished the five T1-like strains from each other were considered (that is, SNPs that distinguished only DC3000 from all five T1-like strains were excluded because they were not informative with respect to evolution of T1-like strains). Trees with identical topology were obtained using only intergenic, intragenic, synonymous, or non-synonymous SNPs (data not shown), suggesting that selection did not significantly affect phylogenetic reconstruction. Typical for recently diverged bacterial genomes [22], no homoplasies or recombination events were detected. Interestingly, strain NCPPB1108 isolated in 1961is located on the most basal branch of the tree, followed by T1 isolated in 1986 on the next branch, while the most recently isolated strains LNPV 17.41 (1996), Max4 (2002), and K40 (2005) cluster together on the most derived branch. This could suggest that in the last 50 years we have witnessed an evolution of T1-like strains whereby the strains found on tomato today may have replaced their ancestors of the recent past and may be relatively more fit.

### A SNP analysis suggests T1-like populations have replaced each other repeatedly over the last 50 years in North America and Europe

**Figure 3:**
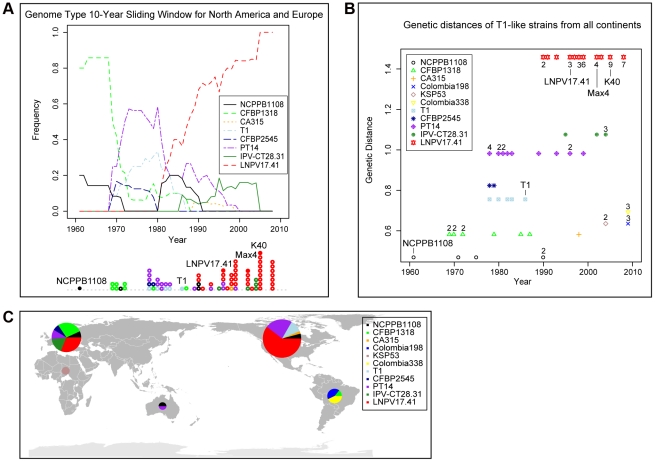
T1 genotypes change in frequency over time and genetic distances from the outgroup strain DC3000 increase over time. Several genotypes are present in both North America and Europe. (A) The lines indicate the frequency of T1 genotypes over time using a 10-year sliding window with a one-year step. Circles represent individual isolates and are placed in the graph in correspondence to the exact year at which isolates were collected. Full circles indicate those isolates for which genomes have been sequenced. (B) Genetic distance of strains from the out-group strain DC3000 plotted over time. Genetic distance was calculated based on the 24 MLST SNPs and the 16 genome SNPs that were analyzed in all strains. When more than one isolate with the same genotype was collected during the same year, the total number of isolates is indicated next to the genotype symbol. (C) World map with pie charts showing ratio of T1 genotypes for the continents from which T1-like strains have been analyzed. Pie size is proportional to the total number of strains considered per continent.

**Figure 4:**
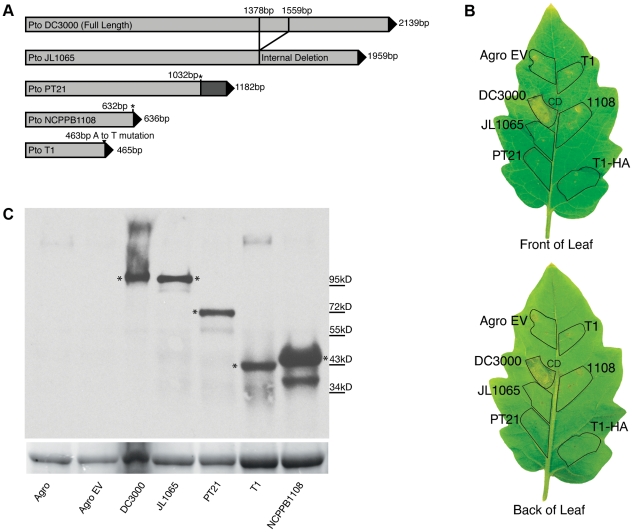
The *hopM1* gene is disrupted in all T1-like and JL1065-like strains. The encoded truncated proteins do not trigger cell death in tomato while the full-length protein encoded by the DC3000 *hopM1* gene does. (A) Graphical presentation of *Pto hopM1* alleles. The stars indicate the position of deletions causing frameshifts in the PT21 and NCPPB1108 alleles. The PT21 allele is present in four strains of SNP genotype NCPPB1108 and in the only strain with SNP genotype CA315 while the NCPPB1108 allele is only present in strain NCPPB1108 (SNP genotype NCPPB1108). The T1 allele is present in all other T1-like strains, which are referred to as T1-proper in the text. (B) Agrobacterium-mediated transient expression of *hopM1* alleles fused to *gfp* in the tomato cultivar “Chico III”. Only the *hopM1*_DC3000_ allele triggered cell death. Similar results were obtained on the tomato cultivars “Rio Grande” and “Sunpride” in at least two independent experiments/cultivar. Leaf areas infiltrated with *Agrobacterium tumefaciens* strains are traced in black. Strain names indicate which *hopM1::gfp* fusion construct was expressed in which leaf area. Agro EV: Agrobacterium carrying an empty vector control, T1-HA: in this leaf area the hopM1_T1_ allele was expressed with an HA tag, CD: cell death. (C) Western Blot analysis with GFP antibody of HopM1::GFP fusion proteins from extracts of *Nicotiana benthamiana* leaf disks infiltrated with the same *Agrobacterium tumefaciens* strains used in panel B. * indicate the bands of the expected size based on the sequence of the *hopM1* alleles in panel A. The Rubisco large subunit band from the Coomassie-stained gel is shown as loading control underneath the Western Blot.

To address the question as to whether T1-like strains have evolved since 1961, we sequenced for all 89 T1-like strains in our collection the seven informative SNP loci distinguishing strains Max4, LNPV17.41, and K40 from strains T1, NCPPB1108, and DC3000 (which were identified in the alignment of the Max4, LNPV17.41, K40, and NCPPB1108 sequencing reads against the T1 genome). We also sequenced for all these strains four of the SNP loci distinguishing strains T1, Max4, LNPV17.41, and K40 from strains DC3000 and NCPPB1108. The analyzed SNPs are highlighted in the Table S2. Eleven different genotypes were identified among the 89 analyzed strains based on these SNP loci and SNPs in the housekeeping genes used for the original MLST analysis. Genotype sequences are listed in Table S4 and genotypes for each strain are listed in Table 1. A maximum likelihood tree was then constructed using DC3000 and JL1065 as outgroup (Figure 2B). When plotting frequency of the identified genotypes over time (Figure 3A), it becomes clear that genotype frequency has changed dramatically since 1961 with different genotypes peaking at different times. Moreover, genetic distance of genotypes appears to be correlated with time since the strains identified in the 1960s and 1970s are more similar to the DC3000 outgroup than the strains isolated during the last 10 years (Figure 3B). This correlation between genetic distance and time was found to be statistically significant for strains collected in Europe, the only continent where strains were consistently sampled between 1961 and 2005. This suggests that genotypes may have evolved from each other. However, the strains from the most basal clade in the tree (Figure 2B) have either a 1 bp deletion or a 5 bp deletion in the gene coding for HopM1, a type III effector known to suppress plant immunity during infection of Arabidopsis by strain DC3000 [23], [24], [25]. These deletions cause frameshifts leading to truncated open reading frames that are respectively 636 and 1182 bp long compared to the full length *hopM1* gene in strain DC3000, which is 2139 bp long (Figure 4A). In contrast, T1-like strains on all other branches of the tree have a *hopM1* allele with a nonsense mutation at bp 463 and the *hopM1* allele of strain JL1065 has a 180 bp long in-frame deletion starting at position 1379. Importantly, besides the 1 bp and 5 bp deletions and the premature stop codon all three *hopM1* alleles present in the T1-like strains have 100% DNA identity to each other including the up-stream promoter region and chaperone gene *shchopM1*. Therefore, three independent mutations truncated *hopM1* very recently in T1-like strains and not even one T1-like strain with the ancestral full-length *hopM1* allele is present in our strain collection. This suggests strong selection for loss of full-length HopM1 (see more below). Interestingly, only six strain out of 89 T1-like strains have the deletions causing frameshifts leading to premature stops at codon 212 and 394 while the other 83 T1-like strains have the *hopM1* allele with the early stop at codon 155. These 83 strains thus represent the main T1-lineage that has been causing bacterial speck since 1969, when the first member of this lineage was isolated in Switzerland. To distinguish the strains belonging to this most common T1 lineage from the other T1-like strains we call these strains from now on “T1-proper”.

The world map in Figure 3C shows that several genotypes within T1-proper are present in North America and Europe, suggesting that these strains have moved with relatively high frequency between continents, possibly within seed shipments. In fact, transmission of *Pto* via infested tomato seed has been documented [26]. Long distance movement of *Pto* through the atmosphere is also a possibility since *P. syringae* bacteria have been isolated from rain and snow [27]. Moreover, as described above, genotypes with increasing genetic distance from the outgroup appear to have replaced one another in North America and Europe. However, members of more ancestral T1 lineages as well as JL1065-like strains have apparently persisted in developing countries in South America, Africa, and Asia (Table 1 and Figure 3). This suggests only occasional movement of *Pto* strains between Europe and North America on one hand and South America and Africa on the other. Moreover, the strains separated from the *Pto* population in North America and Europe seem to continue to adapt to tomato independently as evidenced by mutations found only in these strains (see also results for *fliC* alleles from strains isolated in Colombia below).

### The truncated hopM1 alleles of T1-like strains do not cause cell death

Is it possible that the *hopM1* truncation of T1-proper strains contributed to the worldwide expansion of this lineage? Intriguingly, the full length HopM1 protein of strain DC3000 triggers cell death in several tomato cultivars and wild tomato relatives indicating that it may function as a so-called “avirulence” gene, the product of which is recognized by a plant resistance gene leading to activation of plant defenses including programmed cell death [28]. However, given that mutating *hopM1*_ DC3000_ reduced symptom development during tomato infection and did not increase bacterial population size *in planta*, HopM1_DC3000_ has been considered a virulence factor on tomato [23], [29]. To determine if the truncated *hopM1* alleles that we identified in the T1 and JL1065 lineages lost the ability to trigger cell death in tomato, transient assays expressing all identified *hopM1* alleles directly in tomato leaves using *Agrobacterium*-mediated expression were performed. It was found that the *hopM1*_T1_, *hopM1*_PT21_, *hopM1*_NCPPB1108_, and *hopM1_JL1065_* alleles do not trigger cell death while *hopM1_DC3000_* triggers cell death strongly (Figure 4B). However, when bacterial growth was compared under lab conditions between T1 and a T1 strain expressing *hopM1_DC3000_* ectopically, consistent differences were not observed (data not shown). We thus conclude that full-length HopM1 may be recognized by a tomato resistance gene leading to reduced bacterial growth in field conditions. Alternatively, the cell death triggered by *hopM1_DC3000_* in the *Agrobacterium*-mediated expression assay may not be due to recognition but may be correlated to the known role of *hopM1_DC3000_* in symptom formation [23]. If so, it is possible that the contribution of *hopM1* to disease symptoms may actually lead to an artificial selection against full length *hopM1*: seedlings with severe disease symptoms infected with strains that carry the full length *hopM1* allele may be less likely to be sold to farmers for planting than seedlings with mild symptoms or no symptoms at all that are infected with strains that carry a disrupted *hopM1* allele. Thus, a gene like *hopM1* that increases symptom severity may actually render a plant pathogen less fit in an agricultural setting. Regardless, the obvious selection for inactivation of *hopM1* apparent upon analysis of multiple strains shows how characterization of pathogen populations beyond the study of a single model strain can provide new perspectives on the roles of individual virulence factors.

### Allelic variation among T1-proper strains in the gene fliC

To assess other factors potentially contributing to the success of the T1-proper strains, two additional effector genes, *avrRps4* and *avrPto1*, differing among the five sequenced T1 genomes were analyzed (see Table S5 for results and Table S6 for a list of all predicted effectors in the sequenced T1-like genomes). Neither effector was found to be consistently present or absent in T1-proper strains compared to other T1-like strains indicating that these effectors cannot explain the recent expansion of the T1-proper lineage. Nor was there a correlation with presence or absence of the gene cluster for the biosynthesis of the phytotoxin coronatine, which is known to play an important role in the pathogenesis of strain DC3000 on Arabidopsis [30], or *avrD1*, a gene specifying the production of defense inducing syringolides [31] (Table S5). Also extending the search for differences in gene content beyond known virulence genes did not lead to plausible hypotheses in regard to what might have determined the expansion of T1-proper strains compared to all other *Pto* strains. Only 27 gene families, mostly coding for hypothetical proteins or bacteriophage-related proteins, are present in each of the annotated draft genome sequences of the T1-proper strains T1, K40, and LNPV17.41 but absent from the *Pto* strains NCPPB1108, JL1065 and DC3000 (as determined by using the protein repertoire comparison tool at http://genome.ppws.vt.edu/orthologsorter/).

**Figure 5:**
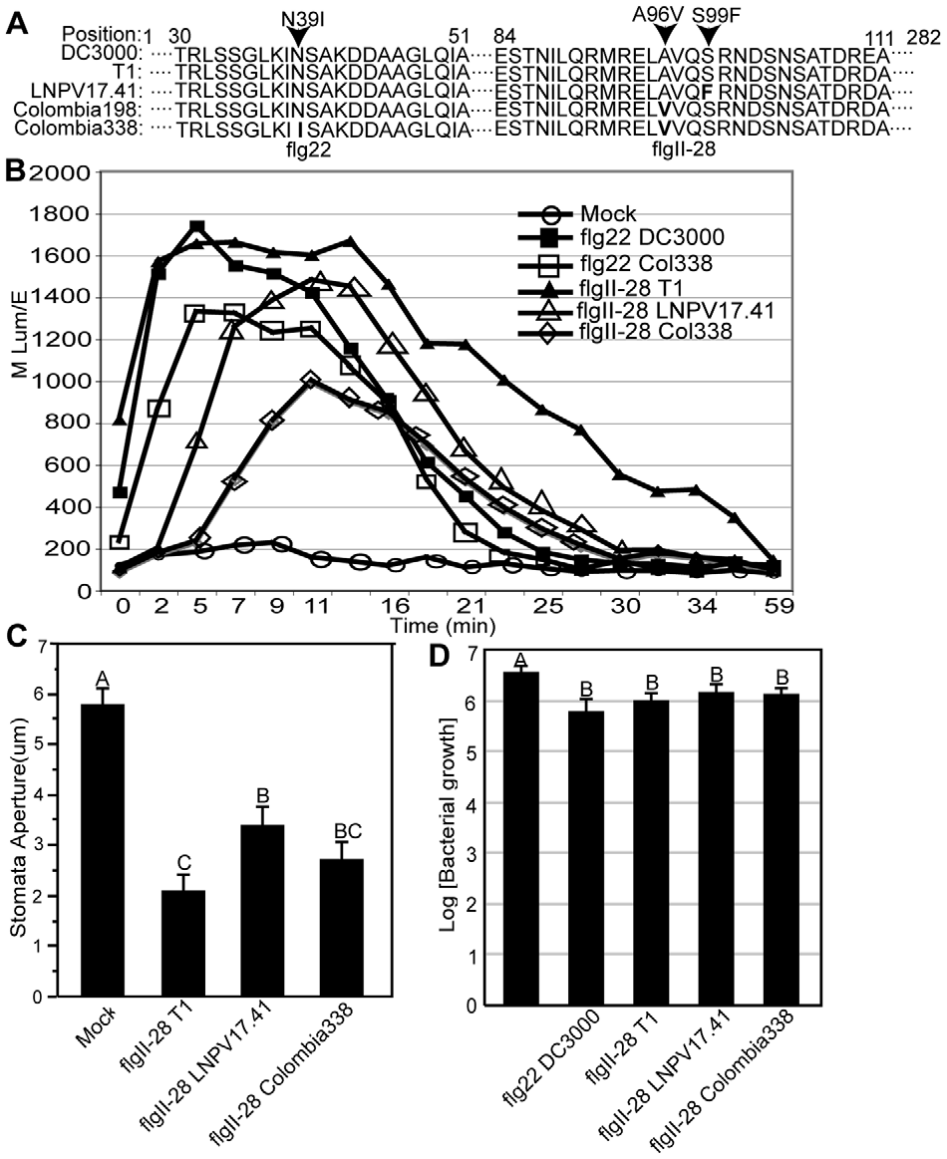
The flagellin epitope flgII-28 triggers reactive oxygen species (ROS) in tomato leaves whereby derived alleles - typical of today's *Pto* strains - induce less ROS than the ancestral alleles - typical of strains isolated before 1985. Alleles of flgII-28 also induce stomatal closure and interfere with leaf invasion. (A) Amino acid sequences of flg22 and flgII-28 alleles. The T1 alleles are identical to the DC3000 alleles and thus represent the ancestral states. The derived alleles are named after one of the genotypes in which they are present. (B) Induction of reactive oxygen species (ROS) in tomato leaf disks of cultivar ‘Chico III’ after incubation with flg22 and flgII-28 peptides at a 1 µM concentration. ROS induction was significantly different at the 2 minutes time point in an unpaired Student's t-test at the 0.05 level between flg22_T1_ and flg22_Colombia338_ and between flgII-28_T1_ on one hand and flgII-28_LNPV17.41_ and flgII-28_Colombia198_ on the other. flgII-28_T1_ and flgII-28_Colombia198_ were also significantly different from each other at the 5 minutes time point. Similar results were obtained with three different tomato cultivars whereby experiments on each cultivar were repeated at least twice. (C) Stomatal closure induced in tomato leaves of cultivar ‘Chico III’ after infiltration with flg22 and flgII-28 peptides at a 5 µM concentration or mock infiltration with sterile water. Similar results were obtained in three independent experiments. Different letters indicate significance at the 0.05 level in an unpaired Student's t-test. (D) Leaves of tomato cultivar ‘Chico III” were infiltrated with flg22 and flgII-28 peptides at a 1 µM concentration. Strain NCPPB1108 (flgII-28_T1_) was then sprayed on leaf surfaces 24 hours later and apoplastic population sizes were measured another 24 hours later. Different letters indicate significance at the 0.05 level in an unpaired Student's t-test.

However, it was striking that one of the seven informative SNPs that distinguished LNPV17.41, K40, and Max4 from T1, NCPPB1108, and DC3000 was in the gene *fliC*, resulting in a S99F mutation (Figure 5A). Intriguingly, the gene *fliC* codes for the flagellum subunit flagellin, well known to contain microbe associated molecular patterns (MAMPs) that trigger an innate immune response in plants and animals [32], [33]. The S99F mutation was found in a majority of T1-proper strains isolated from tomato after 1990 in North America and Europe (see genotypes IPV-CT28.31 and LNPV17.41 in Figure 3). Moreover, of all the mutations analyzed in the 89 *Pto* strains, only this particular SNP was incongruent with other SNPs: the S99F mutation is present in strains KSP53 and KS127M (both of genotype KSP53) from Tanzania, although their genetic background is different from all other strains that carry this mutation. This finding suggests a recombination or parallel evolution event involving *fliC* (which was not detected when sequencing the five T1-like genomes since the genomes of strains 632 and 633 were not completely sequenced) and further supports the idea of strong directional selection on the *fliC* gene. Surprisingly, we even found two additional *fliC* mutations in T1-proper strains belonging to genotypes Colombia198 and Colombia338 isolated in different regions of Colombia in 2008 and 2009. Both mutations are non-synonymous with one of them (D39I) corresponding to a highly conserved amino acid in the middle of the flg22 peptide (Figure 5A), a region of the FliC protein recognized by the tomato immune receptor LeFls2 [34]. The other mutation (A96V) is only two codons away from the *fliC* mutation described above (S99F). These findings suggest that even successful pathogens may be limited in their growth by the plant immune system and to be under selection pressure to further reduce induction of plant defenses. Moreover, the cluster of two mutations in a region apart from flg22 suggests a second region within flagellin besides flg22 that triggers a plant immune response. In fact, infiltrating 28 amino acid long peptides corresponding to the three alternative alleles of this region (denoted as flgII-28), we observed that the ancestral allele triggered induction of reactive oxygen species (ROS) indicative of a plant defense response while ROS triggered by the two derived alleles was significantly reduced and/or delayed depending on the tomato cultivar tested (Figure 5B). The same trend was observed between the ancestral and derived flg22 alleles (Figure 5B). Moreover, infiltration of the ancestral flgII-28 peptide into tomato leaves caused more stomatal closure than infiltration of the derived allele LNPV17.41 (Figure 5C). Stomata are known to be important points of entry into the leaf apoplast for *Pto* [30]. In fact, infiltration of tomato leaves with flgII-28 peptides in advance of spraying bacteria on leaf surfaces reduced apoplastic bacterial population sizes 24 hours after inoculation (Figure 5D). Although the effect of the three different alleles was not significantly different from each other, the ancestral allele consistently reduced population sizes slightly more than the two derived alleles in each of three independent experiments. Taken together, these finding suggest that the mutations in flgII-28 facilitate leaf invasion making strains that carry these mutations more competitive during this important phase of the pathogen life cycle. ROS were also induced by the ancestral flgII-28 allele in *Nicotiana benthamiana* but none of the flgII-28 alleles triggered ROS in Arabidopsis or bean (data not shown). This indicates that flgII-28 is a MAMP, which may be specifically recognized by Solanaceae species. Whether flgII-28 is recognized by the flg22-receptor LeFL2 [34] or if it is recognized by a different receptor remains to be evaluated.

The almost complete worldwide replacement of strains having the ancestral flgII-28 with strains carrying the derived allele highlights how new pathogen variants can rapidly spread around the world. Therefore, reducing movement of plant pathogens between geographic regions represents an important strategy for avoiding spread of increasingly virulent pandemic strains - even in cases when strains or variants of the same pathogen are already present in these regions. Importantly, our data also reveal that MAMPs are more variable than expected. While it was previously reported that strains belonging to the same plant pathogen species can differ in regard to the sequence of the flg22 epitope [35], here we find that even strains belonging to the same clonal lineage can show allelic variation in flagellin. This finding also questions the recently suggested durability of immunity triggered by other MAMPs [36]. However, targeted gene engineering of the *FLS2* receptor gene, and possibly other yet uncharacterized flagellin receptors, may still have potential for strengthening the plant immune response against pathogens with mutated MAMPs.

### Conclusion

We have shown how genome sequencing of multiple isolates of a crop pathogen and analysis of a large collection of isolates with genome-derived markers can yield new insights into plant pathogen evolution and molecular plant-pathogen interactions. We found that the typical bacterial speck pathogen of tomato, represented by the T1-proper lineage, is a recently evolved pathogen that rapidly spread around the world, similar to genetically monomorphic human pathogens like *Yersinia pestis* [1], *Bacillus anthracis* [2], or *Salmonella* Typhi [3]. This suggests that other bacterial plant pathogens may also have adapted to their hosts in recent history, possibly after domestication or - even more recently –after the advent of wide-spread cultivation in mono-culture of their hosts. Investigating microevolution of additional bacterial plant pathogens will make it possible to determine to what point the results obtained here for *Pto* are representative of bacterial plant pathogens in general. Inferring yearly mutation rates and divergence times will be essential for such studies. *P. syringae* pv *aesculi* [21] and *Ralstonia solanacearum* race 3 biovar 2 [37] are examples of plant pathogens that have recently spread to a new world region and for which many isolates collected in recent years from different locations are available. Therefore, these pathogen will be excellent candidates for micro-evolutionary and phylogeographic studies.

Our results also highlight the value of assessing diversity in plant pathogen populations as an important complement to the study of model pathogen strains in lab conditions. This approach can lead to new hypotheses as to why some plant pathogens can cause disease and grow to high numbers on a plant species in lab conditions although they are rarely found on the same plant species in the field while other pathogens are successful both under lab conditions and in the field. Answering this question will be essential for gaining a better understanding of pathogen fitness in the field and to finding new avenues for successful control of plant diseases.

## Materials and Methods

### Bacterial strains, growth and DNA extraction

*P. syringae* pv. *tomato* strains listed in Table 1 were grown in King's Broth (KB) at 28°C and genomic DNA was extracted using the Gentra Puregene Yeast/Bacteria kit (Qiagen) following manufacturer's instructions.

### Multilocus sequence typing

Fragments corresponding to the MLST loci *rpoD*, *pgi*, and *gapA* were PCR amplified and sequenced as previously described [7].

### Genome sequencing

Genomic DNA of strains NCPPB1108, K40, and LNPV 17.41 was sequenced with Illumina technology [9] using the paired-end protocol with read length of 42nt at the University of Toronto Centre for the Analysis of Genome Evolution and Function (CAGEF). Genomic DNA of strain Max4 was also sequenced with Illumina technology but using the single read protocol as previously described for T1 [10]. Genomes of strains NCPPB1108, K40, and LNPV 17.41 were assembled using Velvet 0.7.55 [38]. Insert size for paired-end reads was set to 200; expected coverage was based on the number of reads used in the assembly and the expected genome size based on strain DC3000; coverage cutoff was set to 4; minimum contig length cut off was set to 100. A range of hash sizes was used to obtain the assembly with the highest N50 value and the lowest number of contigs for each genome. Scaffolding was turned off. Genomes were annotated using GRC [39].

### SNP identification

SNPs between *Pto* strain T1 [10] and the other four T1-like strains NCPPB1108, Max4, K40, and LNPV17.41 were identified by aligning Illumina sequence reads of T1, Max4, K40, NCPPB1108, and LNPV 17.41 against the DC3000 genome [8] in MAQ [11]. We only considered the 3,024,986 nucleotides in the DC3000 genome for which there was at least 20X depth of coverage by Illumina reads from each of the five Illumina datasets (i.e. T1, LNPV 17.41, K40, Max4, NCPPB1108) and for which there was at least 95% consensus between the aligned reads. The polymorphism states of the remaining 3,372,140 nt of the DC3000 chromosome were considered to be ambiguous and we made no attempt to detect SNPs there. We considered a SNP to be present at a given site if at least 95% of the aligned reads at that site consistently called a different nucleotide from that in the reference sequence. We compared the position of each SNP against the positions of the predicted genes as specified in RefSeq:NC_004578 to determine whether it was intergenic or intragenic. For intragenic SNPs, we translated the open reading frame containing the SNP to check whether the SNP would result in a different amino acid sequence (i.e. whether it was a non-synonymous mutation). The process was automated using custom Perl scripts. SNPs that were not informative to distinguish T1-like strains from each other were not considered, i.e., all SNPs that distinguished DC3000 from the T1-like strains but that had the same nucleotide in all five T1-like strains. Only the SNP loci that distinguished T1-like strains from each other are shown in Table S1 and were used for construction of the whole genome tree shown in Figure 2A (see below for details).

In a second independent search for SNPs between Pto strains T1, Max4, K40, NCPPB1108, and LNPV 17.41, Illumina sequence reads of the newly sequenced strains were aligned against the T1 draft genome using MAQ [11] using default parameters. The MAQ output was then parsed using a custom script eliminating all SNP calls that did not have the consensus A, C, G or T. A final list of core genome SNPs (Table S2) was then assembled limiting SNPs to SNPs present in genes that were found to be present exactly one time in the *P. syringae* genomes T1 [10], DC3000 [8], B728a [40], and 1448A [41] using OrthoMCL [42]. The total length of these genes is 3,543,009 nt.

### Construction of whole genome trees

Based on silent, non-silent, intergenic, and intragenic sites, we constructed 5 bootstrapped (2000 replicates) Maximum Likelihood trees for the genomes of strains T1, Max4, LNPN17.41, K40 and NCPPB1108 using the genome of strain DC3000 as outgroup. The first four trees were based on each of the data features separately, and the remaining tree was based on the collection of all data features jointly, to which we refer to as the whole genome tree. Trees were constructed in PAUP version 4.0 (http://paup.csit.fsu.edu/) using parameters determined by jMODELTEST [43], [44]. Non-silent, intragenic, and the whole genome data satisfied the GTR substitution model [45]; whereas, silent and intergentic data best fit the GTR+I and SYM models [45], respectively. A Maximum parsimony tree was built using DNAPARS of the PHYLIP 3.69 package (http://evolution.gs.washington.edu/phylip.html).

### SNP analysis

Primers were designed upstream and downstream of each of the seven SNPs that distinguished strains LNPV 17.41, K40, and Max4 from NCPPB1108 and T1. Four primer pairs were designed for additional five SNPs (two of them adjacent to each other) that distinguished LNPV 17.41, K40, Max4, and T1 from NCPPB1108 and DC3000. The 12 SNPs are highlighted in green in Table S2 and primers are listed in Table S7.

### Construction of SNP tree

Based on the SNPs listed in Table S4, 11 genotypes were identified among the T1-like strains listed in Table 1. Table S5 lists the SNP genotype for each strain. jMODELTEST [43], [44] was used to determine the substitution model that best fit the data (SYM). A maximum likelihood tree was then built in PAUP version 4.0 (http://paup.csit.fsu.edu/). Bootstrap analysis was performed with 5000 replicates. A Maximum parsimony tree was built using DNAPARS of the PHYLIP 3.69 package (http://evolution.gs.washington.edu/phylip.html).

### Molecular evolutionary analysis

Based on a 10-year sliding window, we calculated the relative frequencies of T1-, JL1065- and DC3000-like strains, for the time period 1942-2009. Additionally, for the years 1961-2009, T1-like strains acquired across North America and Europe according to genotypes were also analyzed based on a 10-year sliding window. Each T1-like strain was uniquely classified based on a profile of 40 SNPs. Eight genotypes of T1-like strains were observed in North America and Europe. Frequency plots were generated for these genotypes using the statistical software language R (http://www.r-project.org/).

Genetic distances for all T1-like strains were calculated as compared to the DC3000 strain, under the Jukes-Cantor model. In order to investigate the relationship between these relative genetic distances and isolation year, we fit the regression model:
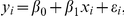
where 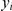 is the relative genetic distance, 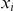 is the isolation year, and 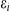 denotes independent normally distributed error. Values of 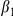 which are distinguishable from zero indicate a linear temporal relationship between genetic distance (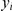) and time (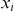).

### Estimation of divergence times

In order to estimate divergence times for the five sequenced T1-like stains (Max4, LNPV17.41, K40, T1 and NCPPB1108), we used IMa2 [17], [18] and BEAST 1.6.1 [19]. In both programs, we computed our estimates based on the nucleotides present at the concatenated SNP loci listed in Table S1 and setting the mutational clock rate (µ) to 1.

IMa2 [17], [18] was run in Markov Chain Monte Carlo (MCMC) mode. We considered our five strains to be derived from five populations, and assumed no migration in the model. The mutation model used for this analysis is the Hasegawa-Kishino-Yano (HKY) model. Prior distributions were selected as uniform distributions between zero and some upper bound. Upper bounds were chosen to be far removed from the maximum likelihood estimate: 300 for 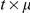, and 200 for effective population size parameters. In order to reduce auto correlations in our MCMC samples, 20 million iterations were run, with samples stored 10,000 iterations after a ‘burn-in’ period of 2 million generations. Multiple runs of the algorithm produced nearly identical results.

In BEAST 1.6.1 [19], prior distributions were selected as lognormal with units in% per million years. GTR was selected as substitution model. Since BEAST results are on a percent scale, results were converted to million years in order to compare to IMa2 results.

To rescale program outputs to an estimated clock rate and to the length of the genome used for SNP discovery, we used:
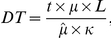
where DT is the rescaled divergence time in years; t is the estimated splitting time obtained from IMa2 or BEAST converted to years; 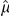 is the mutation rate per base pair (bp) per year; 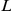 is the length of SNPs used as input, which is 157 bp; and 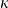 is the total length of the genome used for SNP discovery, which is 3,024,986 bp.

### Effector prediction

Pseudomolecules were created from the draft genome sequences by concatenating contigs in the order from largest to smallest with the TIGR linker sequence “nnnnnttaattaattaannnnn” delimiting contig boundaries. Effectors were identified in the pseudomolecules using a combination of automated annotation generated by RAST (http://rast.nmpdr.org/), alignment of pseudomolecules with the DC3000 sequence visualized using the Artemis Comparison Tool, HrpL binding sites predicted as previously described [46], and PSI-BLAST of confirmed effector sequences against the pseudomolecule sequences. Predicted effectors are listed in Table S6.

### HopM1 cloning and transient expression

The open reading frames including the ribosome binding site but not the stop codon of *hopM1* alleles were amplified by PCR from genomic DNA of *Pto* strains DC3000, JL1065, T1, NCPPB1108, and PT21 with the primer pairs listed in Table S7 and with nested primers to add sequences for Gateway^TM^ (Invitrogen) cloning using the protocol described previously [47]. The five PCR products were then cloned into the entry vector pDNOR207 (Invitrogen) using the Gateway^TM^ BP cloning kit (Invitrogen). Recombined plasmids were confirmed by sequencing and cloned into the destination vector pBAV150 [47] using the Gateway^TM^ LR cloning kit (Invitrogen). *hopM1*-containing pBAV150 were mated from *Escherichia coli* into *Agrobacterium tumefaciens* C58C1 and used in transient assays of tomato leaves (at a concentration corresponding to an optical density at 600 nm of 0.04) and in *Nicotiana benthamiana* leaves (corresponding to an optical density at 600 nm of 0.4) using the same protocol as described previously for *Nicotiana benthamiana* [47]. Western blots were performed as described in [47] also.

### Characterization of MAMP-triggered immunity

Peptides corresponding to alleles of flg22 and flgII-28 were ordered from EZBiolab with >70% purity (see Figure 5 for peptide sequences). Peptides were resuspended in sterile water and used to measure induction of reactive oxygen species (ROS) in the tomato cultivar Chico III. A luminol - horseradish peroxidase assay was used to quantify ROS induction as described by Chakravarthy and colleagues [48] with small modifications: 4-mm leaf discs were punched out with a cork borer and floated adaxial side up in 200 µl ddH2O over night at room temperature in wells of a 96-well solid white plate. The ddH2O was then replaced with 100 µl of ROS testing buffer containing 1 uM of flg22 or flgII-28 peptide, 34 µg/ml of luminol (Sigma), and 20 µg of horseradish peroxidase (VI-A, Sigma). Luminescence was measured using a Bioteck, Synergy HT plate reader. Five leaf disks treated with the same peptide were tested in parallel. Leaf discs in testing buffer without addition of any flagellin peptide were used as a negative controls.

### Analysis of stomatal closure after leaf infiltration with MAMPs

Leaves were treated with flg22 and flgII-28 peptides as described by Melotto and co-workers [30] with slight modifications. Briefly, 4 week-old tomato plants were sprayed with water, placed in transparent plastic bags, and transferred to a 28°C incubator exposed to light to induce stomatal opening. Whole leaves were detached from plants and placed on a glass slide. The leaves were immersed in 5 uM of flagellin peptide dissolved in ddH20, or just ddH2O for mock treatment, and then covered with a cover slip. The mounted leaves were placed at room temperature for 2 hours and then viewed at 200x magnification using an Axio Imager M1 upright microscope (Zeiss). Pictures of stomata were taken using an Axiocam MRm camera (Zeiss). Stomatal aperture of 20 stomata per test group per experiment were quantified using Axiovision v. 4.7.2 (Zeiss).

### Leaf invasion assay

Leaves of 5-week-old tomato plants (cv. ‘Chico III’) were infiltrated with flg peptides at a 1 µM concentration via a blunt end syringe while still attached to the plant. Plants were placed in a high humidity container for 24 hours. Strain NCPPB1108 was then sprayed onto leaves at a concentration corresponding to an optical density at 600 nm of 0.01 in 10 mM MgSO_4_ using a sprayer canister and placed back in the high humidity container. Bacterial invasion was assessed 24 hours after infection. 0.52 mm sections were punched out of the infiltrated leaves and placed in a tube with 200 µL 1% bleach with the leaf punch completely submerged. The tube was mildly vortexed for 5 seconds to remove epiphytic bacteria. The leaf punch was then removed from the 1% bleach solution, gently rinsed in ddH_2_O, and then placed in a separate tube containing 200 µL 10 mM MgSO_4_ and three 2 mm glass beads. The tube was placed in a mini bead beater (Biospec Products, Inc.) and shaken for 90 seconds to grind the leaf and release endophytic bacteria into the solution. Colony forming units were counted after dilution plating.

### Accession numbers

HQ992994 – *hopM1* operon of strain T1

HQ992995 – *hopM1* operon of strain NCPPB1108

HQ992993 – *hopM1* operon of strain PT21

JF268671 - *hopM1* operon of strain JL1065

JF261012 – *fliC* allele of strain K40

JF261011 – *fliC* allele of strain Col198

JF261013 – *fliC* allele of strain Col338

## Supporting Information

Table S1 SNPs identified between Max4, LNPV 17.41, T1, K40, and NCPPB1108 by aligning Illumina reads against the genome of Pto strain DC3000.
(XLS)

Table S2 Core genome SNPs identified between Pto strains T1, Max4, NCPPB1108, K40, and LNPV17.41 by aligning Illumina reads against the T1 draft genome and only considering those SNPs located within core genome genes.
(XLS)

Table S3 Estimation of times in years since most recent common ancestor of T1-like strains with Bayesian 95% Highest Posterior Density intervals assuming a yearly mutation rate per bp of 5×10–6.
(XLS)

Table S4 DNA sequences corresponding to the MLST and SNP genotypes listed in Table 1 (only nucleotides corresponding to SNPs are shown and were used for molecular evolutionary analyses, i.e. nucleotides identical in all analyzed strains were ignored.
(XLS)

Table S5 List of strains with continent and year of isolation, MLST genotype, SNP genotype, and results for several virulence factors based on PCR (and sequencing of PCR products for hopM1).
(XLS)

Table S6 Predicted type III effector repertoires of T1-like strains (positions refer to whole genome shotgun sequences deposited at NCBI, besides Max4. which was not deposited).
(XLS)

Table S7 Primers.
(XLS)
